# Interplay between Fanconi anemia and homologous recombination pathways in genome integrity

**DOI:** 10.15252/embj.201693860

**Published:** 2016-04-01

**Authors:** Johanna Michl, Jutta Zimmer, Madalena Tarsounas

**Affiliations:** ^1^ Genome Stability and Tumourigenesis GroupDepartment of OncologyThe CRUK‐MRC Oxford Institute for Radiation OncologyUniversity of OxfordOxfordUK

**Keywords:** Fanconi anemia, homologous recombination, DNA repair, replication stress, DNA damage response, genome stability, Cancer, DNA Replication, Repair & Recombination, Molecular Biology of Disease

## Abstract

The Fanconi anemia (FA) pathway plays a central role in the repair of DNA interstrand crosslinks (ICLs) and regulates cellular responses to replication stress. Homologous recombination (HR), the error‐free pathway for double‐strand break (DSB) repair, is required during physiological cell cycle progression for the repair of replication‐associated DNA damage and protection of stalled replication forks. Substantial crosstalk between the two pathways has recently been unravelled, in that key HR proteins such as the RAD51 recombinase and the tumour suppressors BRCA1 and BRCA2 also play important roles in ICL repair. Consistent with this, rare patient mutations in these HR genes cause FA pathologies and have been assigned FA complementation groups. Here, we focus on the clinical and mechanistic implications of the connection between these two cancer susceptibility syndromes and on how these two molecular pathways of DNA replication and repair interact functionally to prevent genomic instability.

## Introduction

The Fanconi anemia (FA) family includes 19 distinct functional complementation groups (A, B, C, D1, D2, E, F, G, I, J, L, M, N, O, P, Q, R, S, T) whose gene products suppress interstrand crosslink (ICL) sensitivity. Roles in the repair of other types of DNA damage and in the regulation of replication stress responses have been additionally ascribed. One FA gene subset encodes nine proteins of the FA core complex (FANCA/B/C/E/F/G/L/M/T), which activates the FANCI–FANCD2 heterodimer through monoubiquitination. The remaining eight FA proteins (FANCD1/J/N/O/P/Q/R/S) mediate recombinational and nucleolytic reactions to complete repair (Zhang & Walter, 2014).

Mutations in most FA genes lead to a chromosomal instability disorder characterised by multiple developmental abnormalities, progressive bone marrow failure and cancer predisposition (Kim & D'Andrea, 2012). In many cases, assignment of a gene to the FA family is based on the identification of a small number of patients displaying partial FA pathologies (Table 1). A subset of FA proteins includes the well‐characterised RAD51 recombinase, as well as the tumour suppressors BRCA1 and BRCA2, which are directly involved in the homologous recombination (HR) pathway of double‐strand break (DSB) repair (Venkitaraman, 2009). In this review, we highlight the clinical features of patients carrying mutations in FA and HR genes. We furthermore discuss the roles of FA and HR pathways in the cellular response to exogenous and endogenous sources of DNA damage, and how they impact on telomere and genome integrity.

**Table 1:** FA genes, proteins and pathologies associated with their inactivation

Gene	Synonym	Main protein functions	Gene frequency within FA patient population (%)	Symptoms	References
*FANCA*		Component of FA core complex; interacts with BRCA1	66	FA pathologies	Apostolou *et al* (1996)
*FANCB*		Component of FA core complex	2	FA pathologies	Meetei *et al* (2004)
*FANCC*		Component of FA core complex	10	FA pathologies	Strathdee *et al* (1992)
*FANCD1*	*BRCA2*	HR repair; loads RAD51 onto DNA; interacts with FANCD2 and FANCN; stalled replication fork protection	Rare	FA pathologies; not all patients display bone marrow failure; mutation carriers have higher risk of breast and ovarian tumours and lower onset age	Alter (2006), Howlett *et al* (2002), Wagner *et al* (2004)
*FANCD2*		Ubiquitinated after DNA damage; MCM interaction; stalled replication fork protection	2	FA pathologies	Timmers *et al* (2001)
*FANCE*		Component of FA core complex; interacts with FANCD2	2	FA pathologies	de Winter *et al* (2000)
*FANCF*		Component of FA core complex	2	FA pathologies	de Winter *et al* (2000)
*FANCG*	*XRCC9*	Component of FA core complex	9	FA pathologies	de Winter *et al* (2000)
*FANCI*		Ubiquitinated after DNA damage; activates dormant origins	< 2	FA pathologies	Dorsman *et al* (2007), Sims *et al* (2007), Smogorzewska *et al* (2007)
*FANCJ*	*BACH, BRIP1*	FA repair; HR repair; 3ʹ to 5ʹ helicase; interacts with BRCA1; checkpoint activation	< 2	FA pathologies	Levitus *et al* (2005), Levran *et al* (2005), Litman *et al* (2005)
*FANCL*		E3 ubiquitin ligase; component of FA core complex	Rare	FA pathologies; no cancers reported	Meetei *et al* (2003)
*FANCM*		DNA helicase/translocase; localises the core complex to DNA; required for FANCI–FANCD2 ubiquitination; checkpoint activation	Rare	Phenotype unknown because the only patient described in the literature also has a FANCA mutation	Meetei *et al* (2005)
*FANCN*	*PALB2*	HR repair; promotes BRCA2 function; interacts with BRCA1 and BRCA2	< 2	FA pathologies; mutation carriers have higher risk of breast cancer	Reid *et al* (2007), Xia *et al* (2006a)
*FANCO (provisional)*	*RAD51C*	HR repair; promotes RAD51 nucleoprotein filament stability; ICL repair	Rare	FA‐like syndrome; patients do not thus far display bone marrow failure or cancer	Meindl *et al* (2010), Vaz *et al* (2010)
*FANCP*	*SLX4*	Coordinates XPF–ERCC1, MUS81–EME1 and SLX1 nucleases; resolves Holliday junctions	Rare	FA pathologies	Kim *et al* (2011), Schuster *et al* (2013), Stoepker *et al* (2011)
*FANCQ*	*ERCC4, XPF*	Endonuclease; binds to ERCC1; crosslink unhooking	Rare	FA pathologies; one patient also displayed Cockayne syndrome and xeroderma pigmentosum	Bogliolo *et al* (2013), Kashiyama *et al* (2013)
*FANCR*	*RAD51*	HR repair; ICL repair; protection of nascent strands from DNA2‐ and WRN‐mediated resection; stalled replication fork protection	Rare	FA‐like syndrome; patients do not thus far display bone marrow failure or cancer	Ameziane *et al* (2015), Wang *et al* (2015)
*FANCS*	*BRCA1*	HR repair; promotes RAD51 loading; ICL repair; chromatin dissociation of replicative helicase; stalled replication fork protection; interacts with FANCD2 and FANCN	Rare	FA‐like syndrome; patients do not display bone marrow failure; mutation carriers have higher risk of breast and ovarian tumours and lower onset age	Sawyer *et al* (2015)
*FANCT*	*UBE2T*	E2 ubiquitin‐conjugating enzyme for FANCI–FANCD2 complex; interacts with FANCL	Rare	FA pathologies	Hira *et al* (2015), Machida *et al* (2006), Rickman *et al* (2015), Virts *et al* (2015)

## Pathologies associated with FA and HR gene mutations

FA is a predominantly autosomal recessive disease with an incidence of 1–5 per 1,000,000 births (Auerbach *et al*, 2001). FA patients harbour biallelic mutations in a particular FA gene, with notable exceptions of *FANCB,* which is X‐linked and therefore susceptible to X‐chromosome inactivation, and *FANCR*/*RAD51* (Table 1), in which all identified mutations are dominant negative. FA clinical characteristics include bone marrow failure, developmental abnormalities and an increased risk to develop malignancies. FA diagnosis is confirmed when, in addition to this clinical constellation, ICL hypersensitivity is detected at the cellular level.

Interstrand crosslinks can be induced by a variety of agents, most notably diepoxybutane (DEB), which continues to be used in the clinic as a major FA diagnostic tool (Auerbach & Wolman, 1976), and DNA crosslinking agents used in cancer treatment such as mitomycin C (MMC) and cisplatin. In cells lacking a functional FA pathway, ICLs elicit complex DNA lesions, illegitimately repaired to produce radial chromosomes, which represent the cellular FA signature. These chromosomal aberrations underlie the extreme toxicity of ICL‐inducing treatments to FA cells (Auerbach *et al*, 1989).

At least 20% of FA patients develop cancers (Kutler *et al*, 2003), in particular acute myelogenous leukaemia (AML). However, other tumours including head and neck squamous cell carcinoma, gynaecological squamous cell carcinoma, oesophageal carcinoma, and liver, brain, skin and renal tumours are also associated with FA gene mutations (Alter, 1996; Joenje & Patel, 2001).

It is noteworthy that breast and ovarian tumours rarely occur in FA patients carrying mutations in the core FA genes (Alter, 1996). This may be due to the fact that many FA patients are sterile and probably oestrogen‐depleted. Carriers of monoallelic *FANCC* mutations have only a modest increase in breast cancer risk (Berwick *et al*, 2007). Likewise, *FANCJ* monoallelic mutations rarely predispose carriers to breast and ovarian cancer (Cantor *et al*, 2001; Rutter *et al*, 2003; Seal *et al*, 2006; Rafnar *et al*, 2011). One recent study reported a nonsense variant in *FANCM* (c.5101C>T) associated with a twofold increase in breast cancer susceptibility in the Finnish population (Kiiski *et al*, 2014). A second study reported that the *FANCM* non‐sense mutation c.5791C>T, leading to exon 22 deletion and loss of DNA repair function, also confers a small increase in the familial breast cancer risk (Peterlongo *et al*, 2015). In contrast, mutations in HR genes that have also been assigned to the FA pathway carry a clear risk of breast and ovarian tumour development (discussed below).

### Breast and ovarian tumours associated with mutations in BRCA genes

Monoallelic germline mutations in the tumour suppressor genes *BRCA1* and *BRCA2* predispose women to breast and ovarian cancer (Peto *et al*, 1999). Heterozygous carriers of *BRCA1* or *BRCA2* mutations have a 82% lifetime risk of breast cancer, as well as 54 and 23% risks of ovarian cancer, respectively (King *et al*, 2003). *BRCA1* and *BRCA2* mutations account for approximately 16% of the familial risk of breast cancer (Anglian Breast Cancer Study Group, 2000) and are also associated with increased risk of pancreatic, stomach, laryngeal, fallopian tube and prostate cancer (Venkitaraman, 2009; Roy *et al*, 2012).

Genomic instability caused by defects in DNA replication and repair is the key molecular mechanism underlying cancer predisposition in *BRCA* mutation carriers. *BRCA1/2* heterozygosity is not associated with the overwhelming chromosome instability characteristic of cells with biallelic mutations. Loss of heterozygosity in the affected *BRCA* gene leads to chromosome rearrangements. Subsequent oncogenic events, such as inactivation of tumour suppressors (e.g. *p53*) and/or oncogene induction (e.g. *KRAS*), provide tolerance to chromosomal instability and sustain proliferation under genotoxic stress.

The breast cancer susceptibility associated with *BRCA1/2* deficiencies has been recapitulated in mouse models carrying the respective gene deletions (Evers & Jonkers, 2006). Null mutations in either *Brca* gene are embryonic lethal in mice, with only a mild rescuing effect conferred by concomitant *p53* abrogation. However, studies using a combination of *p53* deletion and conditional *Brca1* or *Brca2* inactivation in skin and mammary gland epithelium (Xu *et al*, 1999; Jonkers *et al*, 2001) have demonstrated prevalent development of mammary tumours. Tumour induction mechanisms other than *p53* deletion are known to potentiate the loss of tumour suppressor functions of *BRCA* genes. For example, a recent study has demonstrated that *Brca2* germline heterozygous mutations are sufficient to promote tumourigenesis in a *Kras* ^*G12D*^ mouse model for pancreatic ductal adenocarcinoma, independently of p53 status (Skoulidis *et al*, 2010).

### Classification of BRCA1 and BRCA2 as FA proteins

Patients with homozygous *BRCA1* or *BRCA2* germline mutations are rare, consistent with these genes being essential for viability. Conceivably, the human mutations reported so far are hypomorphic, with the residual gene expression sustaining survival in the presence of diminished cellular functions. Nevertheless, *BRCA1* and *BRCA2* genes have been assigned FA gene denominations, as *FANCS* and *FANCD1*, respectively (Table 1; Howlett *et al*, 2002; Sawyer *et al*, 2015). The fundamental problem here is the very low number of patients with *BRCA1/2* homozygous germline mutations, who do not live long enough and do not show sufficiently penetrating FA phenotypes to justify this inclusion. For example, the haematological defects or bone marrow failure characteristic of FA are clearly absent in the two patients with homozygous *BRCA1* mutations reported so far (Domchek *et al*, 2013; Sawyer *et al*, 2015).

The first *BRCA1* mutant patient displayed congenital abnormalities, inherited ovarian cancer and carboplatin hypersensitivity, but normal blood count (Domchek *et al*, 2013). Patient death prevented further critical analyses, such as radial chromosome induction by DEB treatment that would have substantiated a *bona fide* FA phenotype. The second patient was a woman diagnosed with multiple congenital anomalies (growth failure, microcephaly and dysmorphic face) indicative of Dubowitz syndrome, who developed breast cancer at age 23 (Sawyer *et al*, 2015). Whole‐exome sequencing revealed distinct *BRCA1* mutations in the two alleles, one of which (c.5095C>T) generates a 35‐amino acids internal deletion and the other a one‐amino acid substitution previously reported to underlie breast cancer susceptibility. Full‐length BRCA1 reconstitution was not performed, although an N‐terminal BRCA1 truncation (BRCA1Δ512‐1283) suppressed DNA damage sensitivity in skin fibroblasts from this patient. Thus, FA pathologies in both cases seem inconclusive and a FA‐like syndrome designation may be more suitable for *BRCA1* mutations (Wang & Smogorzewska, 2015).

The data supporting *BRCA2* classification as a FA gene appear more convincing. The first study that assigned *BRCA2* to the FA complementation group D1 (Table 1) was based on two *BRCA2* homozygous patients with classical FA pathologies, including congenital abnormalities, abnormal skin pigmentation and cellular sensitivity to MMC (Howlett *et al*, 2002). Bone marrow failure or haematological tumours were not detected. However, two more recent studies performed in larger patient cohorts reported that homozygous *BRCA2* mutations are associated with high risk of acute leukaemia during early childhood: 6 out of 7 patients (Wagner *et al*, 2004) and 13 out of 27 patients (Alter, 2006) developed the disease. In *BRCA2* patients, leukaemia was largely refractory to chemotherapy (Wagner *et al*, 2004), suggesting accumulation of additional mutations that obstruct clinical intervention.

Furthermore, haematological defects detected in mouse models for *Brca2* gene inactivation support the latter clinical data. Mice homozygous for a constitutive *Brca2* exon 11 deletion, which abrogates approximately 45% of the *Brca2* transcript, succumb to thymic lymphomas (Friedman *et al*, 1998). A robust hematopoietic defect, albeit without lymphoma development, was reported in mice carrying a homozygous *Brca2* exon 27 deletion (*Brca2* ^*D27/D27*^) (Navarro *et al*, 2006). In addition to spontaneous chromosomal instability in bone marrow cells, these mice show a prominent proliferation defect in hematopoietic progenitors and self‐renewing hematopoietic stem cells.

### Other HR proteins included in the FA pathway

In addition to *BRCA1* and *BRCA2*, other HR genes have been assigned to FA complementation groups (Table 1). Biallelic mutations in PALB2 (partner and localiser of BRCA2, also designated *FANCN)* have been associated with FA clinical features (Levitus *et al*, 2005; Levran *et al*, 2005; Litman *et al*, 2005; Xia *et al*, 2006a; Reid *et al*, 2007). At the molecular level, PALB2 protein bridges the interaction between BRCA1 and BRCA2 in DSB repair (Xia *et al*, 2006b). The PALB2–BRCA1 interaction is regulated by ubiquitination to suppress homologous recombination repair in G1 (Orthwein *et al*, 2015). Importantly, monoallelic mutations in *PALB2* increase the risk of breast cancer (Roy *et al*, 2012).

The *RAD51C* gene, encoding a member of the RAD51 paralog family of HR repair proteins and component of the BRCA2 interactome (Suwaki *et al*, 2011; Reuter *et al*, 2015), has also been implicated in FA. A carrier of a homozygous mutation (c.773G>A) in *RAD51C* leading to a single amino acid substitution was reported to exhibit congenital anomalies characteristic of FA (Vaz *et al*, 2010). However, no bone marrow failure was detected; therefore, *RAD51C* has been provisionally assigned the FA complementation group O (Table 1) and the associated pathology termed an FA‐like syndrome (Kottemann & Smogorzewska, 2013). Moreover, six *RAD51C* monoallelic mutations, which predisposed to breast and ovarian cancer, were identified in German families (Meindl *et al*, 2010). However, the susceptibility of *RAD51C* heterozygous mutation carriers to breast and ovarian cancer has been a topic of debate (Akbari *et al*, 2010; Zheng *et al*, 2010).

One of the newest members of the FA gene family is *RAD51*, which has been assigned the FA complementation group R (Ameziane *et al*, 2015; Wang *et al*, 2015). The first patient identified with a *RAD51* heterozygous mutation featured developmental abnormalities and ICL sensitivity, measured by increased levels of diepoxybutane‐ and MMC‐induced radial chromosomes in peripheral blood lymphoblasts and skin fibroblasts (Wang *et al*, 2015). Lack of bone marrow defects led to classification of the associated disease as an FA‐like syndrome. This *de novo RAD51* heterozygous mutation (c.391A>C) results in a single amino acid substitution, which specifically abrogated ICL repair, whilst recombinational repair remained intact. Mechanistically, the mutant protein acts in co‐dominant‐negative manner (with the wild‐type protein still expressed), triggering extensive DNA2/WRN‐dependent resection (see below) and hyper‐phosphorylation of Replication Protein A (RPA). Characterisation of this FA patient is a remarkable illustration of an entirely novel concept, namely that naturally occurring separation‐of‐function mutants enable distinction between ICL versus DSB repair roles of factors previously believed to exclusively play roles in HR. More recently, a second patient carrying a distinct dominant‐negative heterozygous mutation in *RAD51* (c.877G>A) has been identified (Ameziane *et al*, 2015). This patient's clinical pathologies included primarily aberrant neurological functions. Sensitivity to crosslinking agents could be detected at the cellular level; however, HR repair capacity has not been investigated.

## The FA and HR pathways of DNA repair

### The current model of ICL repair

The FA repair pathway is required for genome protection against ICLs. This specific type of DNA damage, considered to be amongst the most deleterious DNA lesions, obstructs both replication and transcription (Kee & D'Andrea, 2010). ICLs can be induced by chemotherapeutic agents (e.g. cisplatin, MMC), which are used as non‐specific DNA damage inducing agents in the clinic. Additionally, acetaldehyde and formaldehyde—aldehyde by‐products of cellular metabolism—have been identified as endogenous ICL sources that require FA proteins for repair (Langevin *et al*, 2011; Pontel *et al*, 2015). Thus, disruption of FA genes in normal cells leads to accumulation of ICL‐induced replication‐associated damage, mutations and chromosomal aberrations, which underlie the pathologies associated with FA.

**Figure 1:**
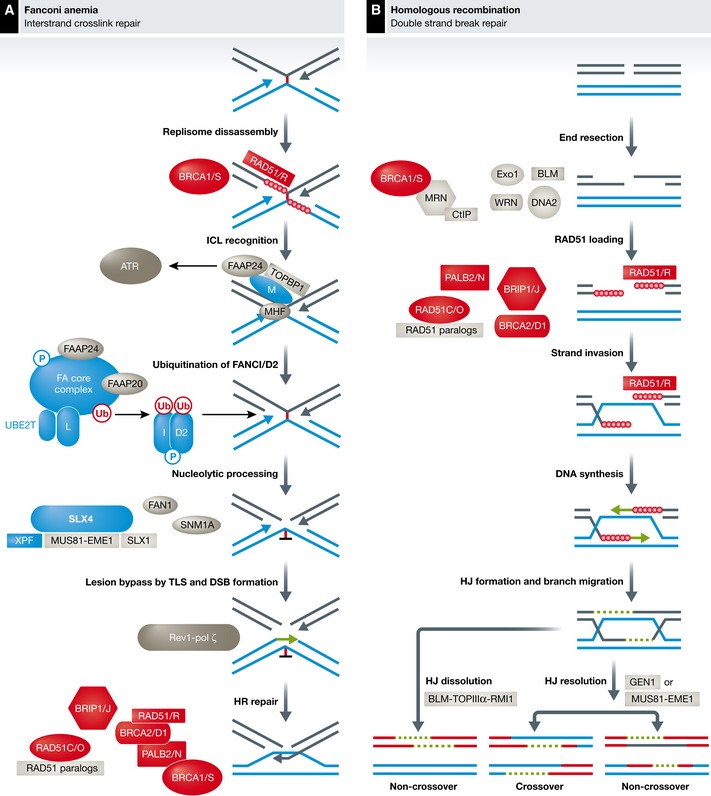
Interstrand crosslink (ICL) and double strand break (DSB) repair pathways (A) The Fanconi anemia (FA) pathway of ICL repair. Upon fork stalling at ICL sites, BRCA1 acts to dismantle the replisome (not shown) and RAD51 binds to the single‐stranded DNA to protect the fork. Subsequent FANCM–FAAP24–MHF1/2 complex binding activates ATR signalling and promotes recruitment of the FA core complex. The core complex in turn ubiquitinates the FANCI–FANCD2 heterodimer, which acts via SLX4 as a platform to recruit multiple nucleases (ERCC1‐XPF, SLX1 and MUS81‐EME). Nucleolytic incisions unhook the ICL and facilitate translesion synthesis‐dependent lesion bypass, mediated by REV1 or Polζ polymerases. The thus‐generated DSB is repaired by HR. (B) The HR pathway of DSB repair. DNA ends at a break site are resected to generate single‐stranded DNA tails. Resection is initiated by the MRN complex, stimulated though CtIP interaction and further extended though the activities of EXO1, BLM, WRN and DNA2. The resulting single‐stranded DNA is a substrate for RAD51 monomer loading in BRCA2‐ and RAD51 paralog‐dependent manner. The nucleoprotein filament thus generated invades a homologous double‐stranded DNA and, following second‐end capture, a double Holliday junction structure is generated. Branch migration facilitates cleavage of Holliday junctions by GEN1 or SLX4‐MUS81‐EME1‐SLX1 resolvases, or their dissolution dependent on the BLM–TOPIIIα–RMI1 complex. Crossover or non‐crossover molecules are the final products of the DNA repair reaction. Blue, FA proteins; red, HR proteins annotated as FA complementation groups; grey, other proteins associated with each pathway.

The most recent model for ICL repair (Fig 1A) (Zhang & Walter, 2014) suggests that two convergent replication forks collide at an ICL site. This implies that the DNA surrounding the lesion is already replicated when the block is encountered and replication restart is not required. According to this widely accepted model, ICL repair is elicited when the replisome is partially dismantled by eviction of MCM replicative helicase subunits from the chromatin, thereby enabling ICL recognition by FANCM and its interacting partners FAAP24 and MHF1/2 (Ciccia *et al*, 2007; Collis *et al*, 2008). FANCM binding adjacent to ICLs leads to recruitment of the core FA complex and ATR‐dependent checkpoint activation, which stalls the replisome. The binding of the FA core complex to the lesion triggers monoubiquitination of the FANCI–FANCD2 complex, as the central event in the FA pathway. Monoubiquitinated FANCI–FANCD2 is recruited to the chromatin and orchestrates downstream reactions including endonucleolytic incision, translesion synthesis and DSB repair. Additionally, FANCI–FANCD2 SUMOylation has recently been reported to occur in response to DNA damage in ATR‐ and FA core‐dependent manner (Gibbs‐Seymour *et al*, 2015). This important posttranslational modification is known to coordinate various aspects of the DNA damage response, in concert with ubiquitination (Jackson & Durocher, 2013). In the case of FANCI–FANCD2 complex, SUMOylation regulates its eviction from chromatin to limit incision at the ICL site.

Six different endonucleases have been implicated in ICL repair: MUS81‐EME1, SLX1‐SLX4, XPF‐ERCC1 (FANCQ), Fanconi‐associated nuclease 1 (FAN1), SNM1A and SNM1B. Whether the last two function in FA‐dependent manner is unclear (Zhang & Walter, 2014). SLX4 functions as the scaffold protein for incision nucleases and may be recruited by monoubiquitinated FANCI–FANCD2 (Fig 1A; Kim *et al*, 2011). However, subsequent work has shown that SLX4 recruitment to ICLs precedes FA core complex binding and FANCI–FANCD2 ubiquitination (Raschle *et al*, 2015). The main function of SLX4 is to recruit XPF‐ERCC1, the key nuclease for the direct incision that unhooks the crosslink (Klein Douwel *et al*, 2014). In addition to XPF‐ERCC1, the nucleases SLX1, MUS81‐EME1 and FAN1 have redundant functions in introducing incisions, and their interaction with SLX4 is required to various degrees for ICL repair (Kim *et al*, 2013). The resulting lesion on one sister chromatid is bypassed by translesion DNA synthesis, dependent on REV1 and DNA polymerase ζ (Budzowska *et al*, 2015), concomitantly with removal of the unhooked adduct by nucleotide excision repair. The DSB generated on the second sister chromatid is most commonly repaired by HR, although other repair pathways can also be engaged (see below).

Studies using *Xenopus* egg extracts have established important roles for HR factors during early stages of ICL processing, prior to DSB formation. Fork stalling in the proximity of ICLs triggers dissociation of the replicative helicase to enable lesion processing (Fu *et al*, 2011). The single‐stranded DNA generated in this way must be “primed” for subsequent HR repair reactions (Long *et al*, 2011). Consistent with this notion, RPA and RAD51 bind in proximity of ICLs before DSB formation, and act there to prevent fork breakage or degradation and to promote regulated incisions. Human RAD51 also acts to protect ICL‐stalled forks against nucleolytic degradation (Wang *et al*, 2015). Whether BRCA2 mediates loading of RAD51 at these sites, as it does at DSBs, has not yet been determined.

In addition to RAD51, BRCA1 also functions early in ICL repair, before the incision stage (Long *et al*, 2014). In hydroxyurea (HU)‐treated mammalian cells, BRCA1 plays fork protection roles, a function shared by RAD51, BRCA2 and FANCD2 (Schlacher *et al*, 2011, 2012; Hashimoto *et al*, 2012). In *Xenopus* egg extracts, BRCA1 is additionally required to unload the replicative GMC (GINS, CDC45, MCM2‐7) helicase (Ilves *et al*, 2010), rendering the surrounding chromatin conducive for ICL repair reactions (Long *et al*, 2014). However, complementation of the BRCA1‐depleted extracts by BRCA1–BARD1 does not restore unloading, suggesting that additional factors/modifications are necessary.

### The HR pathway of DSB repair

DSBs represent key intermediates in ICL repair. It is therefore anticipated that one of the two major DSB repair pathways, HR or non‐homologous end joining (NHEJ), is involved in the final steps of ICL repair. NHEJ provides an error‐prone mechanism for the repair of DSBs, which is active throughout the cell cycle, whilst HR reactions occur primarily in S and G2 when a sister chromatid is available for use as repair template. The complex interplay between NHEJ and FA factors, as well as the contribution of NHEJ to ICL repair in cells lacking a functional FA pathway, has been discussed in detail elsewhere (Kottemann & Smogorzewska, 2013). In this review, we focus on HR, known to provide the major mechanism for repair of replication‐associated DNA damage, including crosslink repair (Tsang & Carr, 2008; Aze *et al*, 2013). Moreover, HR factors assemble at ICL sites during early stages of damage processing. Therefore, it is likely that most DSB intermediates in ICL repair are channelled into the HR pathway.

The HR repair reaction involves three major steps (Fig 1B; Tacconi & Tarsounas, 2015): DSB end resection, strand invasion and Holliday junction resolution. Resection is initiated by the MRE11–RAD50–NBS1 (MRN) complex, together with the interacting partner CtIP, and is further extended through concerted activities of exonuclease 1 (Exo1), Bloom's syndrome RecQ helicase‐like protein (BLM), Werner syndrome ATP‐dependent helicase (WRN) and DNA replication ATP‐dependent helicase/nuclease 2 (DNA2) (Mimitou & Symington, 2011; Nimonkar *et al*, 2011). The 3′ overhang thus generated is stabilised by RPA binding. BRCA2 recruitment facilitates active removal of RPA, concomitant with RAD51 loading onto the single‐stranded DNA overhangs. The RAD51 paralog family also plays a role at this stage, possibly by stabilising the RAD51 nucleoprotein filaments and/or promoting their invasion into a homologous, intact double‐stranded DNA. Following second‐end capture and DNA synthesis, a double Holliday junction structure is formed. Branch migration promotes Holliday junction cleavage by GEN1 or SLX4‐MUS81‐EME1‐SLX1 resolvases, or junction dissolution mediated by the BLM–TOPIIIα–RMI1 complex.

## The roles of FA and HR in the replication stress response

While it is clear that FA and HR pathways are strongly linked to each other genetically, the precise molecular mechanisms underlying the functional interactions between HR and the FA proteins in normal cell physiology remain to be elucidated. During unchallenged cell cycle progression, FA and HR proteins fulfil repair‐independent functions, regulating the cellular responses to endogenous replication stress. Failure of these functions leads to mutations, chromosome rearrangements, which drive FA pathologies and HR‐loss‐induced tumorigenesis.

**Figure 2:**
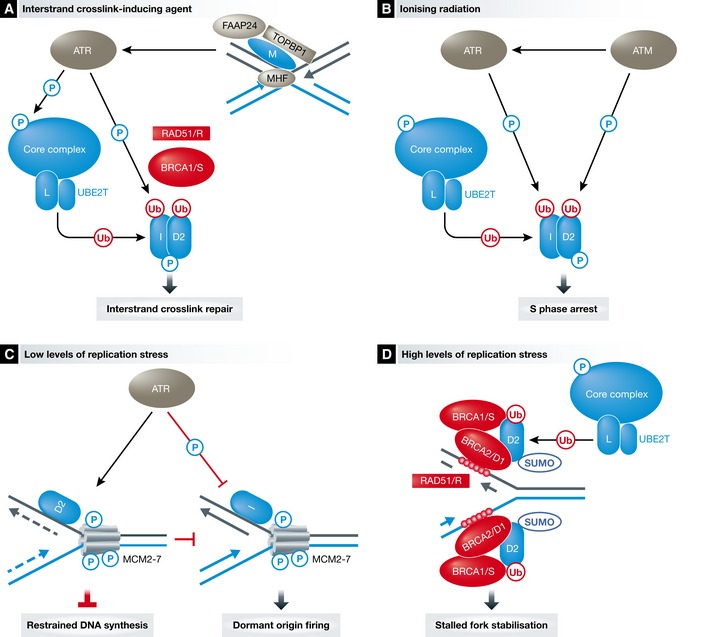
Fanconi anemia (FA) pathway activation in response to DNA damage and replication stress (A) ICL‐induced fork stalling recruits the FANCM–FAAP24–MHF1/2 complex, which, in turn, activates ATR signalling. ATR phosphorylates components of the FA core complex (FANCA and FANCE) and FANCI–FANCD2. FA core complex recruitment to damage site leads to FANCI–FANCD2 monoubiquitination and chromatin binding to initiate repair. (B) IR‐induced DNA damage elicits ATM and ATR activation leading to phosphorylation of FANCD2. ATR is required for the efficient monoubiquitination of FANCD2 by the core complex, which triggers cell cycle arrest. (C) Low‐dose (e.g. 0.5 mM) HU treatment elicits ATR activation and FANCD2 binding to MCM2‐7, which limits DNA synthesis. Concomitantly, FANCI also binds the MCM complex to promote dormant origin firing. ATR‐dependent FANCI phosphorylation inhibits dormant origin firing and initiates DNA repair/replication fork restart. FANCD2 also inhibits FANCI‐mediated dormant origin firing, independently of its monoubiquitination status. (D) High‐dose (2–5 mM) HU treatment elicits activation of the classical FA pathway. BRCA1, BRCA2 and monoubiquitinated FANCD2 are recruited to stalled replication forks to protect them from degradation by stabilising RAD51 filaments on single‐stranded DNA.

In order to achieve high‐fidelity duplication of the genome, the replisome must overcome barriers arising not only at sites of DNA crosslinks introduced by endogenous aldehydes, but also at DNA secondary structures such as G‐quadruplexes (G4s), RNA–DNA hybrids (R‐loops) or stable protein–DNA complexes. Replication forks frequently stall at these sites, leading to aberrant replication fork structures which accumulate single‐stranded DNA and elicit replication stress responses (Zeman & Cimprich, 2014). To study replication stress *in vivo*, replication is perturbed using replication‐stalling agents, for example HU (Fig 2). FA and HR proteins share several protective functions against replication failure, including regulation of origin firing and replication fork restart, stabilisation and protection of stalled replication forks against nucleolytic degradation. These, together with additional functions specific to FA (e.g. replication fork remodelling, unwinding of G4 DNA) or HR factors (e.g. repair of DSBs arising at sites of stalled replication), are discussed below.

### BRCA1 and BRCA2

A role for BRCA2 in stabilising stalled forks was first reported by Venkitaraman and colleagues in 2003 (Lomonosov *et al*, 2003). This study reported that Y‐shaped DNA junctions that identify stalled replication forks detected in 2D gel electrophoresis disappear during HU‐induced replication arrest in BRCA2‐deficient mouse cells. The explanation for this puzzling observation came from a later study (Schlacher *et al*, 2011), which used DNA fibre analyses to demonstrate that in the absence of BRCA2 stalled forks are degraded in MRE11‐dependent manner. Mutational analysis revealed that the conserved BRCA2 C‐terminus, required to stabilise RAD51 filaments, but not to load RAD51 onto DNA, was essential for the protection of stalled forks. A subsequent study also implicated FANCD2 and BRCA1 in this fork protection mechanism (Schlacher *et al*, 2012).

BRCA1 and BRCA2 are also known to preserve genome integrity by repairing R‐loop‐associated DNA damage. R‐loops are three‐stranded structures, consisting of a RNA:DNA hybrid plus the DNA coding strand, known to identify replication fork barriers. BRCA2‐depleted cells accumulate R‐loops, suggesting a role for BRCA2 in their processing (Bhatia *et al*, 2014). A similar function for BRCA1 was recently reported (Hatchi *et al*, 2015). Genome‐wide analyses revealed BRCA1 enrichment at R‐loop‐rich termination regions of actively transcribed genes, where it facilitates senataxin recruitment to provide a mechanism for repair of R‐loop‐induced DNA damage.

G‐quadruplexes or G4‐DNA are DNA secondary structures that obstruct replication fork progression and pose a threat to genome integrity. BRCA1‐ and BRCA2‐compromised cells are hypersensitive to G4 accumulation, highlighting the role for HR in the repair of G4‐associated DNA damage (Zimmer *et al*, 2016). G4 stabilisation with chemical ligands (e.g. pyridostatin) triggers excessive levels of replication stress, particularly toxic in the context of BRCA1/2 deficiency. This provides means for selective targeting of *BRCA1/2*‐mutated cells and tumours.

### FANCI and FANCD2: coordinated and independent functions

FANCI and FANCD2, central components of the FA pathway, play key roles in the cellular response to replication stress (Fig 2C and D). A replication function for FANCD2 was first proposed in 2005, based on the observation that FANCD2 monoubiquitination in response to HU or aphidicolin treatment blocks processive DNA synthesis (Howlett *et al*, 2005). FANCD2 is required for protection of replication forks stalled in the presence of HU (Schlacher *et al*, 2012), further substantiating a FANCD2 function in replication stress responses.

Using isolation of proteins on nascent DNA (iPOND) combined with mass spectrometry, FANCD2 and FANCI were identified as replisome‐associated factors in HU‐arrested cells, specifically bound to the nascent DNA (Lossaint *et al*, 2013; Sirbu *et al*, 2013). FANCD2 interacts directly with MCM helicase subunits to limit DNA synthesis in the presence of reduced nucleotide pools (Lossaint *et al*, 2013). FANCI also interacts with MCMs, but promotes dormant origin firing under low replication stress conditions (Fig 2C; Chen *et al*, 2015b). In addition to HU‐induced replication arrest, FANCI and FANCD2 are required for common chromosomal fragile site stability in response to aphidicolin treatment (Howlett *et al*, 2005; Chan *et al*, 2009). Importantly, the two proteins co‐localise to damaged chromosomal fragile sites on mitotic chromosomes in response to replication stress, suggesting that chromatin recruitment is essential for their function (Chan *et al*, 2009; Naim & Rosselli, 2009).

Recent studies identified R‐loops as an endogenous substrate for activation of the FA pathway under physiological conditions. FANCD2 and FANCA abrogations (García‐Rubio *et al*, 2015; Schwab *et al*, 2015) lead to R‐loop accumulation. Inhibition of transcription or enzymatic degradation of R‐loops through RNaseH1 overexpression rescued replication fork arrest and DNA damage accumulation in FA‐compromised cells (Schwab *et al*, 2015), establishing R‐loops as an important source of replication stress in these cells.

Most of the studies outlined above focused on FANCD2‐deficient cells. It is not clear, however, whether these phenotypes can be extended to cells lacking FANCI. According to current models (Fig 1A), the FANCI–FANCD2 complex binds to DNA upon ICL induction and becomes monoubiquitinated by the FA core complex via the E3 activity of FANCL. A recent study identified a module of FANCL, FANCB and FAAP100 as the minimal sub‐complex required for FANCI–FANCD2 ubiquitination *in vitro* (Rajendra *et al*, 2014). Moreover, ubiquitination of either FANCI or FANCD2 is essential for the maintenance of ubiquitin on the other (Smogorzewska *et al*, 2007). FANCD2 ubiquitination is required to prevent R‐loop accumulation (Schwab *et al*, 2015) and nucleolytic degradation of stalled forks (Schlacher *et al*, 2012). It is therefore conceivable, although not yet demonstrated, that FANCI plays a similar role in these processes. However, a variety of recent studies demonstrated that FANCI and FANCD2 fulfil several of their functions independently of each other and of monoubiquitination by the FA core complex (discussed below).

### FANCD2‐independent functions of FANCI

Although both FANCD2 and FANCI have by iPOND been found to associated with replisomes after HU treatment, only FANCI was detected at active replication forks already prior to fork stalling (Sirbu *et al*, 2013). This suggested the possibility that FANCI plays FANCD2‐independent roles in the replication stress response. For example, FANCI promotes dormant origin firing in response to low levels of replication stress, whilst FANCD2 suppresses it (Chen *et al*, 2015b). Interestingly, this function of FANCI is inhibited by ATR‐dependent phosphorylation and is independent of the canonical FA pathway (Fig 2C). Likewise, FANCI function in ICL repair is independent of its monoubiquitination by the FA core complex, because complementation of FA‐I patient cells with the FANCI K523R ubiquitination mutant rescued the MMC sensitivity of these cells (Smogorzewska *et al*, 2007).

Whilst the roles of FANCI in replication seem to be FA core‐independent, FANCI itself is critically required for FA pathway activation in response to replication stress. FANCI, but not its partner FANCD2, acts to recruit the FA core complex to sites of DNA damage (Castella *et al*, 2015). FA core complex recruitment does not require monoubiquitination or ATR‐dependent phosphorylation of FANCI, but is dependent on USP1‐mediated FANCI deubiquitination. Collectively, these results support the view that FANCI activation and its cellular roles are far more complex than originally anticipated.

### FANCI‐independent functions of FANCD2

HU‐induced FANCD2 interaction with the MCM2‐MCM7 replicative helicase is critical for FANCD2‐dependent replisome surveillance and does not require FANCD2 ubiquitination (Lossaint *et al*, 2013). This is therefore a FANCD2 function independent of FANCI and the FA core complex. Moreover, FANCD2 K561R ubiquitination‐defective mutant can suppress origin firing during low‐dose HU treatments (Fig 2C) at similar levels to its wild‐type counterpart (Chen *et al*, 2015b). In contrast, high‐dose HU elicits FA‐ and FANCD2‐dependent monoubiquitination of FANCI to promote replication fork protection and/or restart of stalled forks, suggesting a concerted action of FANCI and FANCD2 under severe replication stress conditions (Fig 2D).

FANCD2, but not FANCI, is a key regulator and interacting partner of the BLM helicase (Chaudhury *et al*, 2013). Consistent with a FA pathway‐ and FANCI‐independent function, non‐ubiquitinated FANCD2 recruits BLM and its interacting partners RMI1, TOP3a and RPA1‐3 to chromatin to restart stalled forks and suppress new origin firing. Upon HU exposure, FANCD2 forms a complex with RAD51 and RAD18, the E3 ubiquitin ligase responsible for PCNA sliding clamp monoubiquitination and translesion synthesis activation, again independent of FANCD2 monoubiquitination (Chen *et al*, 2015a).

### FANCM

The *FANCM* gene encodes a DNA helicase/translocase, proposed to scan the DNA for ICLs and to recruit the FA core complex for ICL repair (Fig 1A). Consistent with this, FANCM is required for FANCD2 phosphorylation and ubiquitination, a downstream event in the FA pathway (Meetei *et al*, 2005; Mosedale *et al*, 2005). Additionally, FANCM can also function independently of the FA core complex to activate ATR signalling in response to replication stress. This role requires the ATPase domain, but not the translocase domain of FANCM (Collis *et al*, 2008; Huang *et al*, 2010). Further substantiating this unique role of FANCM, the FA core components are not required for ICL‐induced RPA recruitment to the chromatin, a critical step for checkpoint activation (Huang *et al*, 2010). The FANCM stress‐induced checkpoint function is mediated by FANCM‐dependent chromatin recruitment of TOPBP1, an essential ATR co‐factor (Schwab *et al*, 2010). Failure to retain TOPBP1 on the chromatin leads to a defect in phosphorylation of downstream ATR targets, including CHK1 and SMC1. Interestingly, CHK1 and FANCM protect each other from proteasomal degradation during DNA replication stress (Luke‐Glaser *et al*, 2010), probably mediated by a direct interaction between FANCM and the checkpoint kinase.

In the presence of replication inhibitors that cause low levels of DNA damage (e.g. aphidicolin), FANCM counteracts fork movement, possibly by remodelling fork structures (Luke‐Glaser *et al*, 2010). In contrast, at sites of damage, FANCM promotes replication fork restart (Luke‐Glaser *et al*, 2010; Schwab *et al*, 2010). In cells lacking FANCM, the progression of replication forks is accelerated, suggesting that FANCM controls DNA chain elongation in the absence of exogenous sources of DNA replication stress. These data have led to the proposal that FANCM constitutively binds to DNA, acting as a DNA lesion sensor and activating the S‐phase checkpoint to recruit the FA core complex. This, in turn, enables repair of damage and restart of stalled replication (Luke‐Glaser *et al*, 2010; Schwab *et al*, 2010). In addition, FANCM effectively dismantles R‐loops, transcription intermediates known to interfere with DNA replication (Schwab *et al*, 2015). This is mediated by the robust DNA translocase activity intrinsic to FANCM, with high affinity for a variety of branched DNA molecules including those whose single‐stranded DNA is bound by RPA (Gari *et al*, 2008).

### FANCJ

FANCJ, a RECQ‐like helicase with 5′ to 3′ directionality, was initially identified as BACH1 (BRCA1‐associated C‐terminal helicase; also known as BRIP1; Cantor *et al*, 2001) required for HR repair of IR‐ or HU‐induced DSBs (Litman *et al*, 2005). FANCJ is required for FANCD2 loading onto the chromatin and FANCD2 foci formation in response to MMC (Zhang *et al*, 2010; Chen *et al*, 2014), but paradoxically, it is not implicated in FANCD2 monoubiquitination (Litman *et al*, 2005). Additional studies are required to clarify whether FANCJ functions downstream of this critical step in FA repair.

Similarly to FANCI and FANCD2, FANCJ acts at the interface between DNA damage repair and replication stress. FANCJ foci assemble spontaneously during S‐phase progression; upon HU‐induced replication stress, they co‐localise with PCNA (Zhang *et al*, 2010). The recruitment of FANCJ to RPA‐containing replication foci is dependent on its helicase activity and its ability to interact with BRCA1 (Gupta *et al*, 2007). *In vitro*, RPA stimulates the helicase activity of FANCJ. Importantly, FANCJ also interacts with TOPBP1, a factor required for ATR checkpoint activation. Both TOPBP1 interaction and FANCJ helicase activity are required for RPA chromatin accumulation and checkpoint activation (Gong *et al*, 2010). Taken together, these results suggest that FANCJ promotes checkpoint signalling in response to replication stress, possibly through unwinding and exposing single‐stranded DNA at stalled replication forks.

Characterisation of Dog‐1, the *C. elegans* ortholog of mammalian FANCJ, provided the first evidence that FANCJ can act to resolve G4 DNA structures (Cheung *et al*, 2002; Youds *et al*, 2007). During normal development, *dog‐1* mutant worms evict extensive guanine‐rich tracts from their genome, which were subsequently shown to contain the G4 signature (Kruisselbrink *et al*, 2008). Consistent with a role in G4 resolution, human FANCJ, a structure‐specific 5′–3′ DNA helicase, can unwind G4 DNA *in vitro* (London *et al*, 2008; Wu *et al*, 2008). Importantly, FA‐J patient cells accumulate large genomic deletions in the proximity of sequences with high G4‐forming potential, reminiscent of the worm phenotype. *In vitro*, the mutant protein form of FANCJ expressed in these patient cells was also unable to resolve G4s (London *et al*, 2008).

The function of FANCJ in G4 stability was strengthened by subsequent studies in DT40 chicken cells, where FANCJ plays a dual role: it mediates epigenetic stability in the proximity of G4s by coordinating the action of REV1 translesion polymerase with BLM/WRN helicases (Sarkies *et al*, 2012) and it promotes processive DNA synthesis and maintenance of chromatin structure at G4 sites (Schwab *et al*, 2013). Recently, the role of FANCJ in resolving G4s during eukaryotic DNA replication was reconstituted in *Xenopus* egg extracts (Castillo Bosch *et al*, 2014).

Telomeres have well‐established G4‐forming potential due to their G‐rich repetitive sequence. It is therefore surprising that FANCJ does not appear to have telomeric functions. In *C. elegans*, telomere length is unaffected in *dog‐1* mutants. This could be due to the low G4‐forming potential of telomeric DNA sequence in worms, which consist of TTAGGC repeats (Cheung *et al*, 2002). In human cells, treatment with the G4‐stabilising compound telomestatin, known to cause telomere dysfunction, induces apoptosis in FANCJ‐depleted cells (Wu *et al*, 2008). However, the contribution of telomere dysfunction to this compound toxicity has not been evaluated. Surprisingly, a recent study reported that *Fancj* ^*−/−*^ mouse cells lack sensitivity to G4‐stabilising compounds (Matsuzaki *et al*, 2015), which seems to contradict biochemical and *in vivo* data supporting a role for FANCJ in G4 resolution.

## Crosstalk between FA and HR in DNA repair

The integrated action of FA and HR pathways in the maintenance of genome integrity was initially established through co‐immunoprecipitation and co‐localisation studies, although follow‐up data to strengthen such findings are still missing for several of these cases (see below). Upon exposure to ionising radiation, HU or MMC, monoubiquitinated FANCD2 is targeted to nuclear foci containing BRCA1, BRCA2 and RAD51 (Garcia‐Higuera *et al*, 2001; Taniguchi *et al*, 2002; Hussain *et al*, 2004; Wang *et al*, 2004; Nakanishi *et al*, 2005). One study reported that FANCD2 and BRCA2 co‐immunoprecipitate from MMC‐treated human and hamster cells, but this observation requires further validation (Hussain *et al*, 2004). Likewise, the IR‐induced FANCD2 interactions with BRCA1 (Garcia‐Higuera *et al*, 2001) or the MRN subunit NBS1 (Nakanishi *et al*, 2002) represent potentially important preliminary observations, which, however, are yet to be confirmed by independent studies. Whilst recombinational repair of IR‐induced DNA damage is well documented, whether the FA pathway proper plays a role in the IR response remains unclear. Consistent with this notion, FA cells are only mildly sensitive to IR (Kalb *et al*, 2004), probably because the HR machinery remains intact.

Conversely, however, it is well established that ICL‐inducing treatments are toxic to HR‐deficient cells. In particular, BRCA1‐ and BRCA2‐deficient cells are highly sensitive to cisplatin and MMC (Patel *et al*, 1998; Moynahan *et al*, 2001; Rottenberg *et al*, 2007). This sensitivity is exploited in the clinic through selective targeting of *BRCA1*/2‐deficient tumours with ICL‐inducing agents, including MMC and cisplatin (Powell *et al*, 2002). The major drawback of such therapies is that acquired secondary mutations restore the wild‐type BRCA1/2 reading frame or enable other mechanisms of resistance to platinum‐based chemotherapy (Sakai *et al*, 2008; Bouwman & Jonkers, 2012). MMC and cisplatin treatments are likewise toxic to RAD51‐ and RAD51 paralog‐deficient cells (Godthelp *et al*, 2002; Ohashi *et al*, 2005; Meindl *et al*, 2010; Jensen *et al*, 2013; Wang *et al*, 2015).

The hypersensitivity of HR‐compromised cells to ICL‐inducing treatments may stem from loss of recombinational repair of DSBs arising during ICL processing. Alternatively, HR deficiency may interfere with other key steps of the FA pathway. For example, BRCA1 inactivation in *Xenopus* egg extracts prevents removal of the replicative helicase and subsequent loading of ubiquitinated FANCD2 in the vicinity of ICLs (Fig 2A; Long *et al*, 2014). Consistent with this, FANCD2 foci formation is impaired in mouse *Brca1* ^*D11/D11*^ cells after exposure to DNA crosslinking agents (Bunting *et al*, 2012). Thus, toxic ICL accumulation resulting from inadequate FANCD2 recruitment underlies the hypersensitivity of BRCA1‐deficient cells to crosslinking agents. Whether the same is true for BRCA2 deficiency remains to be determined. It is conceivable that BRCA2 promotes RAD51 loading onto ICL sites during early steps of ICL processing (Long *et al*, 2011; Wang *et al*, 2015). Although not yet formally demonstrated, an early role for BRCA2 in ICL repair would be consistent with the ICL sensitivity of BRCA2‐deficient cells and tumours.

It is noteworthy that FA patients cells are generally not sensitive to poly(ADP‐ribose) polymerase (PARP) inhibitors (Kim *et al*, 2013), although a broader spectrum of toxicity against FA cell lines had initially been suggested (Gaymes *et al*, 2008). Only mutations in genes with basic HR roles (*FANCD1/BRCA2*,* FANCN/PALB2*,* FANCR/RAD51* and *FANCS/BRCA1*) are notably hypersensitive to PARP inhibitors (Kim *et al*, 2013).

## The role of FA and HR pathways at telomeres

Telomeres are specialised structures that cap the ends of chromosomes, thereby preventing their recognition as DSBs. Telomere dysfunction triggered by telomere shortening and fusions between short or uncapped telomeres can lead to genomic instability. Whether telomere dysfunction can contribute significantly to the genomic instability, characteristic of FA cells, has not yet been established. Moreover, unaltered telomeres were reported in human and mouse FANCG‐deficient cells (Franco *et al*, 2004) and in *Fancj*‐deleted mouse cells (Matsuzaki *et al*, 2015). Likewise, no effect of *Fancc* deletion was detected in mouse cells with normal telomerase activity (Rhee *et al*, 2010). Only when *Fancc* was abrogated in a mouse model lacking telomerase activity, short telomeres showed higher levels of recombination, suggesting that FANCC may suppress such events when telomerase is abrogated.

Surprisingly, FA patient cells show some telomere shortening. The comparison is most frequently to non‐isogenic normal controls and therefore not entirely reliable. It is generally accepted, however, that telomere shortening in FA is due to stem cell failure and increased cell proliferation, but not to an intrinsic telomere maintenance defect. This is contrary to dyskeratosis congenita, another bone marrow failure syndrome, where the telomere shortening is far more profound and emanates directly from severely damaged telomeres (Alter *et al*, 2015).

Consistent with a role in telomerase‐independent telomere maintenance, the FA pathway is involved in telomere length maintenance through the alternative lengthening of telomeres (ALT), a mechanism known to act in cells in which telomerase activity is compromised. FANCD2 co‐localised with telomeres in ALT cells, but not in non‐ALT cells, in manner dependent on FANCL and FANCA (Fan *et al*, 2009), factors that also sustain FANCD2 monoubiquitination.

In contrast to FA, HR factors including RAD51, RAD51 paralogs and BRCA2 are required for telomere replication and capping (Tacconi & Tarsounas, 2015) and thus the genomic instability characteristic of HR‐deficient cells and tumours may have a telomere dysfunction component. Cells lacking the HR factors RAD51C, BRCA2 and RAD51 have short telomeres, as a result of unrepaired DSBs and loss of telomeric DNA. Telomere breakage is thought to emanate from telomere replication defects, as telomeres constitute intrinsic barriers to replication fork progression due to their heterochromatic structure and G4‐forming potential. Consistent with this notion, HR‐deficient cells display elevated levels of fragile telomeres (Badie *et al*, 2010). More recently, fragility of telomeric G‐rich strand with G4‐forming potential was detected in HR‐deficient cells, supporting the HR role in facilitating replication of telomeric G4 structures (Zimmer *et al*, 2016).

Similarly to HR factors BRCA1 and BRCA2, FANCD2 acts to protect stalled replication forks genome‐wide (Schlacher *et al*, 2011). It is therefore conceivable that FA and HR proteins could act together at telomeres to prevent replication‐induced telomere damage. Thus, the pathologies characteristic of FA‐ and HR‐compromised cells are likely caused in part by a telomere replication defect.

## Future perspectives

The interplay between FA and HR pathways in DNA repair and replication is crucial for the maintenance of genome integrity. A better understanding of the fine mechanistic details of their interactions will enable not only a better understanding of DNA repair in general, but will also open new opportunities for clinical applications. In particular, this may be relevant to ICL‐inducing platinum‐based chemotherapies routinely used in breast and ovarian cancer treatment, including *BRCA*‐deficient cancers. The major problem with these drugs is that most patients become resistant to them, which leads to tumour relapse. Reactivation of the FA pathway in these tumours may be one of the resistance mechanisms. If this proves to be the case, current approaches to target FA deficiency can be redeployed to the chemotherapy‐resistant *BRCA1/2*‐mutated patient subset.

Importantly, cancer treatments for FA patients are limited, as all cells of FA patients are as sensitive to DNA‐damaging agents as the cancer cells. Thus, most of the currently used treatments specific to BRCA1/2‐deficient tumours are likely to be very toxic to FA patients, which creates an enormous problem. Genetic alterations specific to the cancer cells need to be identified in order to allow specific targeting of tumours, without highly toxic/potentially lethal side effects for the patient.

The physiological functions of FA and HR pathways are not completely understood. Recent research provided a glimpse into how both pathways facilitate replication through cell‐intrinsic obstacles, including ICLs, fragile sites, G4s and R‐loops. Unravelling the full range of sources of endogenous damage that activate the two pathways represents a challenge for the future. With new technologies advancing fast, it becomes possible to identify genomic locations where damage is likely to arise upon specific loss of FA or HR factors. These, in turn, may be informative on the source of damage and enable better management of the associated pathologies.

## Conflict of interest

The authors declare that they have no conflict of interest.
